# Atrial Fibrillation in a Young Patient Using High-dose Oral Diclofenac: A Case Report

**DOI:** 10.5811/cpcem.52890

**Published:** 2026-03-29

**Authors:** Sabri Onur Çağlar, Hilal Çağlar, Serdar Hira

**Affiliations:** * Bandırma Royal Hospital, Department of Cardiology, Balıkesir, Türkiye; † Bandırma Royal Hospital, Department of Physical Therapy, Balıkesir, Türkiye; ‡ Bandırma Royal Hospital, Department of Biochemistry, Balıkesir, Türkiye

**Keywords:** case report, diclofenac, atrial fibrillation, pain

## Abstract

**Introduction:**

Diclofenac sodium is a widely used medication for its analgesic and anti-inflammatory properties. Although the adverse effects of diclofenac are well described, diclofenac-associated new-onset atrial fibrillation in a young, healthy adult has not been previously reported.

**Case Report:**

A 22-year-old man with severe pain following an ankle injury used diclofenac sodium at a dose of 50 mg orally three times daily for one week. At the end of the week, he presented to the emergency department (ED) complaining of palpitations for a few hours. The patient had no past medical history. His physical examination revealed no pathologic signs except for tachycardia and an irregularly irregular pulse rate. An electrocardiogram showed an irregularly irregular rhythm with a ventricular rate of 128 beats per minute (bpm) (rapid ventricular response) and absent P waves, consistent with atrial fibrillation. The patient was monitored in the ED, and a standard regimen of intravenous amiodarone was initiated for rate control. Approximately three hours after initiation, sinus rhythm returned and his heart rate stabilized at 60 bpm.

**Conclusion:**

This case report describes a possible association between diclofenac use and new-onset atrial fibrillation in a young, previously healthy individual. The findings highlight the need for awareness of the potential arrhythmogenic effects of diclofenac and underscore the importance of taking a careful drug history in patients presenting with unexplained atrial fibrillation.

## INTRODUCTION

Diclofenac sodium is a nonsteroidal anti-inflammatory drug (NSAID) used to relieve pain by inhibiting cyclooxygenase (COX) enzymes, reducing the production of prostaglandins, precursors to inflammatory molecules.^1^ It is widely available over the counter in low-, middle-, and high-income countries and is even prescribed to patients with known cardiovascular comorbidities, under the assumption that low doses are safe, although this has not been proven. While the side effects of diclofenac are well known, there are few reports of diclofenac-induced atrial fibrillation in young patients with no previous cardiac history.^2^,^3^ Here we report a young patient with new-onset atrial fibrillation that may have been related to high-dose oral diclofenac sodium.

## CASE REPORT

**Image 1:**
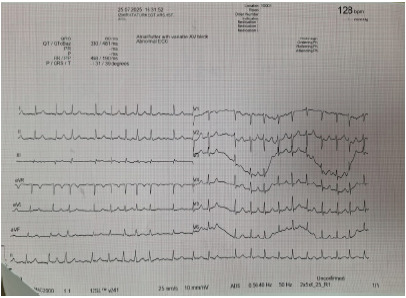
Electrocardiogram of a 22-year-old male who used high-dose oral diclofenac for pain, demonstrating atrial fibrillation with rapid ventricular response.

A 22-year-old male experienced severe pain after spraining his ankle while playing football. He used diclofenac-sodium at a dose of 50 mg orally three times daily for a week. At the end of the week, he presented to the emergency department (ED) complaining of palpitations for a few hours. The patient had no past medical history. He did not take any regular medications and further reported no use of alcohol, caffeine, stimulants, or drugs. After physical examination by the emergency physician the patient was then referred to a cardiologist. No pathologic signs were observed except for tachycardia and an irregularly irregular pulse rate. An electrocardiogram showed an irregularly irregular rhythm with a ventricular rate of 128 beats per minute (bpm) (rapid ventricular response) and absent P waves, consistent with atrial fibrillation (Image 1).

**Image 2:**
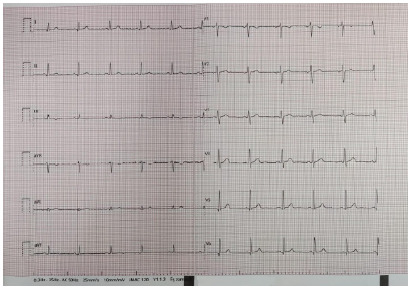
Electrocardiogram showing restoration of normal sinus rhythm after administration of intravenous amiodarone.

Cardiac point-of-care ultrasound demonstrated a preserved global and segmental left ventricular systolic function without valvular abnormalities or intracardiac thrombus. Laboratory results, including complete blood count and routine biochemical tests, were within normal limits. The patient was admitted to a monitoring bed, and the standard regimen of amiodarone was initiated: a 150 mg intravenous bolus over 10 minutes, followed by 900 mg (6 ampoules) diluted in 500 mL of 5% dextrose and infused over 24 hours—1 mg per minute for the first six hours and 0.5 mg per minute for the next 18 hours. Approximately three hours into the infusion, sinus rhythm returned and heart rate decreased to 60 bpm; the infusion was then discontinued to avoid further bradycardia (Image 2). Biochemical tests, including troponin and creatine kinase-MB, were monitored to evaluate for acute coronary syndrome and remained within reference ranges. During the 24 hours of observation, the patient had no additional cardiac problems and was discharged uneventfully with treatment recommendations.

## DISCUSSION

Diclofenac has analgesic, anti-inflammatory, antipyretic, and anti-cancer effects and is widely used for the treatment of various conditions, including painful musculoskeletal disorders, dysmenorrhea, fever, and arthritis, as well as cancer and neurodegeneration.^4^ Diclofenac is mainly associated with the occurrence of gastrointestinal side effects involving abdominal pain, diarrhea, dyspepsia, nausea, and gastrointestinal reflux with long-term use; other observed side effects include hepatic and renal toxicity.^1^ Cardiovascular toxicity has also become a growing concern, particularly with long-term or high-dose exposure.^1^ Even in individuals without pre-existing cardiovascular disease, diclofenac has been associated with a small but measurable increase in events such as myocardial infarction, stroke, and thromboembolism.^2^

*CPC-EM Capsule*

What do we already know about this clinical entity?

*Atrial fibrillation is a common arrhythmia often associated with structural or metabolic disorders*.

What makes this presentation of disease reportable?

*This case describes atrial fibrillation in a young patient following high-dose oral diclofenac use*.

What is the major learning point?

*Non-cardiac medications such as NSAIDs may trigger arrhythmias even in young individuals without comorbidities*.

How might this improve emergency medicine practice?

*Awareness of drug-related arrhythmias can help emergency physicians identify and manage unexpected atrial fibrillation cases promptly*.

Atrial fibrillation is the most common arrhythmia whose prevalence has increased globally, particularly with age.^5^ It is known to contribute to heart failure, cerebrovascular events, coronary artery disease, and higher incidence of mortality.^6^ Some medications, such as corticosteroids, have been associated with an increased risk of atrial fibrillation, but data on the possible role of diclofenac in the development of AF remain sparse.^7^ Okuyan reported paroxysmal atrial fibrillation developing 20–30 minutes after intramuscular diclofenac administration in a 48-year-old patient.^3^ Epidemiological evidence indicates that initiating diclofenac is associated with an increased risk of developing cardiovascular disease, including atrial fibrillation or flutter, when compared with no drug use, paracetamol, or other conventional nonsteroidal anti-inflammatory drugs (NSAID), particularly within the first 30 days of treatment initiation.^2^

Large, population-based studies support this hypothesis. A Danish nationwide analysis found that both non-selective and COX-2 selective NSAIDs were linked to a transient rise in atrial fibrillation incidence during the first month of treatment.^8^ Similarly, a meta-analysis found a 1.2-fold increased risk of atrial fibrillation in users of non-aspirin NSAIDs compared to non-users, and this risk increased by 1.5-fold in new users, suggesting that this may represent a class effect rather than an isolated drug phenomenon. The study also found that COX-2 inhibitors, particularly diclofenac, were associated with higher risks than non-selective NSAIDs.^9^

The precise mechanisms linking diclofenac use to atrial fibrillation are still not fully understood. Several hypotheses have been proposed based on the known pharmacological properties of NSAIDs. Diclofenac exerts strong inhibition of COX-2, which alters prostaglandin and thromboxane synthesis and may impair vascular homeostasis and endothelial function, thereby promoting atrial remodeling and electrical instability.^10^ Experimental data indicate that diclofenac can modulate cardiac electrophysiology by inhibiting voltage-gated potassium and calcium channels and altering repolarization dynamics, which may shorten atrial refractory periods and facilitate re-entry mechanisms.^11^ Furthermore, diclofenac has been shown to increase oxidative stress and systemic inflammation, processes that contribute to structural atrial remodeling and arrhythmogenesis.^12^,^13^ Volume retention and blood pressure elevation associated with NSAID use can also increase atrial wall tension and stretch, further facilitating abnormal conduction and ectopic activity.^8^

Taken together, these mechanisms suggest that diclofenac may increase susceptibility to atrial fibrillation through both direct electrophysiological changes and indirect systemic effects, particularly in predisposed individuals or during high-dose exposure. The current case highlights this possibility given the short-term use of high-dose oral diclofenac before the onset of atrial fibrillation in a previously healthy young adult. While the close timing of drug intake and symptom onset supports a possible temporal relationship, definitive conclusions about causality cannot be drawn from a single report. Additionally, given the relatively short time course of conversion (~3 hours) with amiodarone administration, spontaneous conversion of paroxysmal atrial fibrillation cannot be excluded. Large-scale studies and meta-analyses have demonstrated an association between NSAID exposure and atrial fibrillation risk, particularly in populations with cardiovascular comorbidities. Our observation provides further insight into this association.

## CONCLUSION

This case highlights the need for awareness of the potential arrhythmogenic effects of commonly used NSAIDs such as diclofenac and underscores the importance of taking a careful drug history in patients presenting with unexplained atrial fibrillation. Further epidemiological and mechanistic studies are needed to clarify whether this association reflects a true causal relationship or is a coincidental occurrence.
